# Standardized Communication Across Interprofessional Teams: A Quality-Improvement Initiative to Support Nursing Staff Retention

**DOI:** 10.3390/nursrep16080265

**Published:** 2026-07-30

**Authors:** Vanessa Cameron, Sara Neil, Jamie Jones-Winer, Brittany Justice, Mary Wackerlin, Kristie Young, Madeline Paliganoff, Morgan Nutt, Jessica Snapp, Rachel Kromer, Sandra F. Simmons

**Affiliations:** 1 Nursing Education Professional Development, Vanderbilt University Hospital, Vanderbilt Health, Nashville, TN 37203, USA; jamie.jones-winer@vumc.org (J.J.-W.); brittany.c.justice@vumc.org (B.J.); mary.l.wackerlin@vumc.org (M.W.); kristie.kendall@vumc.org (K.Y.); rachel.kromer@vumc.org (R.K.); 2 Department of Enterprise Analytics, Vanderbilt Health, Nashville, TN 37232, USA; sara.schuster@vumc.org (S.N.); jessica.snapp@vumc.org (J.S.); 3 Department of Strategy and Improvement, Vanderbilt University Hospital, Vanderbilt Health, Nashville, TN 37232, USA; madeline.paliganoff@vumc.org (M.P.); morgan.nutt@vumc.org (M.N.); 4 Division of Geriatrics, Center for Quality Aging, Department of Medicine, Vanderbilt University Medical Center, Nashville, TN 37212, USA; sandra.simmons@vumc.org

**Keywords:** professional delegation, workforce, patient care team, interprofessional relations, interprofessional education, quality improvement

## Abstract

**Background/Objectives**: The purpose of this nursing-led quality-improvement initiative was to strengthen organizational culture, support staff well-being, and improve patient safety by implementing an evidence-based collaboration model that redefined registered nurse (RN) and unlicensed assistive personnel (UAP) teamwork in acute care settings. **Methods**: Guided by transformational leadership principles, Nursing Professional Development (NPD) practitioners, nurse directors and managers, and frontline staff collaboratively redesigned communication and workflows. After piloting the process change, expansion occurred across acute care and step-down units through leader-supported team training alongside ongoing rounding, observations, and feedback cycles. The four phases of the EPIS framework of implementation science were used to implement the initiative, and data were collected via direct observation, anonymous staff surveys, and organizational retention and quality reports. **Results**: Across the seven units in Cluster 1 of the expansion, 335 RNs and 180 UAPs participated. Standardized communication using a structured handover tool increased from 18% to 95% with corresponding improvements in discussions of quality-related measures such as fall risk, turn schedules, and toileting. Patient safety measures exceeded threshold goals for fiscal year (FY) 2026 across falls with harm, pressure injuries, and catheter-associated urinary tract infections (CAUTI). RN and UAP retention demonstrated overall improvement, with RNs exceeding their retention goal and UAPs exceeding their goal by removing a single outlier unit. Perceptions of overall team communication throughout the shift increased for both RNs and UAPs. **Conclusions**: This initiative exemplifies how uniting transformational leadership with frontline staff empowerment supports inclusion, improved quality measures, and strengthened workforce resilience. This scalable framework for nurse-led transformation fosters belonging, safety, and shared outcomes across organizations.

## 1. Introduction

The US Bureau of Labor Statistics anticipates growth in the role of registered nurses (RNs) of 5% from 2024 through 2034, which is above average compared to other occupations [1]. There are approximately 189,000 openings projected annually through 2034, with most of these openings replacing workers who leave the profession either due to career changes or retirement [1]. Similarly, the American Association of Colleges of Nursing (AACN) recognized intensification of the RN shortage due to both the retirement of active nurses and a growing need for healthcare providers within communities [2]. While there are many contributing factors, such as faculty shortages impacting nursing school enrollment rates and an aging population, the additional stress level of nurses in the post-pandemic work setting is a contributing factor to nurses leaving the profession [2].

Numerous studies have demonstrated that higher RN staffing is associated with better patient outcomes. With fewer RNs to manage the same level of patient care, there is evidence of worsening patient outcomes, including hospital-acquired infections and deaths, as well as increased rates of burnout among staff [3,4,5,6]. A Press Ganey report in 2024 identified that higher RN turnover was directly linked to increased rates of inpatient hospital patient falls and incurred more expenses for the healthcare organization [7].

Collaboration with unlicensed assistive personnel (UAPs) is identified as a critical component in the future of healthcare delivery [8]. Increased presence of UAPs, including working at the top of their scope, can reduce the workload of nurses and improve budgetary concerns for hospitals [8]. However, patient safety remains the top priority as the skill mix, or RN and UAP ratio, changes [9]. Nursing staff handoff communication has been identified as a critical component of patient safety, yet UAP staff may lack the experience and knowledge to recognize care priorities or critical information [9]. Standardizing handoff and prioritizing safety information in the presence of all relevant team members can improve patient safety and quality measures [9]. While UAP turnover has not been directly studied in relation to patient outcomes, the skill mix of the interprofessional team to support patient safety is clear.

Recognizing current and expected future nurse staffing challenges, a large adult hospital adjusted the skill mix of nursing teams on acute care and step-down units to effectively utilize both RNs and UAPs, organizationally referred to as Care Partners. This collaborative team approach required greater RN–UAP communication to facilitate effective teamwork. Literature findings indicate that standardized communication between RN and UAP roles and intentional formal team training will positively influence these factors [8,9]. The purpose of this quality-improvement initiative was to increase RN and UAP retention, strengthen organizational perceptions and direct behaviors of teamwork, and improve patient safety by implementing an evidence-based collaborative model that redefined RN and UAP partnerships in acute care settings.

## 2. Methods

This quality-improvement initiative included collaboration with frontline staff and unit leaders. The implementation team consisted of the Nursing Professional Development (NPD) director, NPD practitioners, Human Resources Business Partner, data analyst, operations improvement engineer, and project manager. Those in NPD roles support ongoing professional education for nurses and other healthcare professionals, in many systems being referred to as “educators” or other role titles. This work was identified by the Vanderbilt IRB to be a non-research quality-improvement initiative in the IRB submission #231168. It did not meet the criteria for research as described in 45 CFR 46.102(I), where “research means a systematic investigation, including research development, testing and evaluation, designed to develop or contribute to generalizable knowledge.” The designation of non-research quality improvement waives the required informed consent in accordance with 45 CFR part 46 and in alignment with internal organizational IRB policy and procedure.

This organization identified a significant concern regarding UAP turnover rates, with a pre-implementation 12-month retention rate of 57.8% within the identified acute care and step-down hospital units. Compounding known RN shortages and high UAP turnover worsens clarity and understanding of individual and team responsibilities, creates poor task delegation, and exacerbates challenges in team communication [10]. Recognizing UAP retention as an organizational priority led to the completion of a literature review of factors that influence UAP retention. This review, completed in January 2024 and synthesized in April 2024, identified a wide variety of UAP retention strategies, with the top two work environment influences being interpersonal relationships [11,12] and teamwork, including task delegation [11,13]. UAPs who felt empowered, respected, valued, and involved in their daily work and teams had less intent to leave [11]. Building meaningful interpersonal relationships contributes to a positive workplace culture [12], and improving interpersonal relationship efforts must also include effective team functioning to support ongoing learning and growth alongside respect and trust [11,13].

Further exploration into teamwork and effective delegation between UAPs and RNs demonstrated the importance of evidence-based team training [14], daily team communication [15], and the use of assessment tools to identify gaps and recognize progress with UAP and RN teamwork functioning [15,16,17]. Interprofessional team members found team training better supported professional behaviors, knowledge, engagement with content and colleagues, and general attitudes [14], all of which supported positive teamwork, communication, and patient safety [14,17]. Intentional daily communication between team members increased overall levels of communication throughout the day between team members and job satisfaction and perceptions of teamwork [15]. For evaluating teamwork and communication, studies demonstrate the value of using a standardized assessment tool [15,16,17]. Many tools are based on perceptions of teamwork [17], but this does not adequately capture missed care or the ability to effectively communicate care priority actions between team members [16]. Behavior observation tools were recognized as one method to accurately capture data related to team functioning and communication [18].

The literature review findings that guided this work have been supported by more recent publications. A 2025 scoping review found five critical themes to effective team functioning between RNs and UAPs: role clarity, delegation, communication, unit culture and practice, and interpersonal relationships [10]. Identified challenges that prohibit effective teamwork between RNs and UAPs included lack of role clarity and expectations, poor delegation skills, and interprofessional communication [10]. One identified opportunity to more effectively engage RNs and UAPs in teamwork and communication to support patient care was the inclusion of UAPs in a structured, interprofessional shift handover [10]. Moreover, through strong interpersonal relationships and effective team functioning, it is possible to retain high-quality measures, encourage all team members to work at the top of their scope, and improve retention of staff [8].

### 2.1. VRCP Program

Using the EPIS Framework of implementation science, the four phases of Exploration, Preparation, Implementation, and Sustainment were completed [19]. The Exploration Phase focused on the literature reviews and synthesis of findings. This information was taken to key invested groups, such as nursing executive leadership, shared governance groups, and the Patient Family Advisory Council, to discuss the relevance of findings and opportunities for effective integration into the current environment.

The Preparation Phase was guided by the literature findings on strategies for UAP retention and effective teamwork, leading to the development and implementation of three unique team management tools: (1) standardized communication expectations and processes, (2) evidence-based team training to support these processes, and (3) staff observations and surveys for ongoing monitoring. This intervention bundle became the foundation of the Vanderbilt RN and Care Partner Partnership (VRCP). The purpose of the intervention bundle was to support the integration of the new communication workflows into frontline practices and strengthen perceptions of teamwork with a primary goal of improved UAP retention.

The Implementation Phase occurred initially across six acute and step-down care units as part of a pilot program to determine the feasibility and impact of this initiative, refine standardized communication tools, and complete inter-rater reliability (IRR) with trained observers for behavior observation for data collection. During the pilot phase, implementation was successful across all pilot units. Ongoing observations for adherence, in addition to frontline staff surveys, were conducted throughout the three months following implementation to support the Sustainment Phase. However, opportunities in the quality improvement implementation were identified and integrated. Changes made between the pilot and future implementation phases included adjustments to the VRCP Reliability Checklist to reduce the number of “not applicable” opportunities, refinements to the formal team training content and engagement strategies, focusing on comparing current and future state, and increased emphasis on the team-based process change rather than a change primarily driven by UAPs. The IRR process reviewed 48 distinct observation fields and was achieved through 21 observations across 4 days, with IRRs between observers ranging from 96 to 98%, meeting the IRR goal of 95%. Nursing-sensitive quality indicators of falls, pressure injuries, and catheter-associated urinary tract infections (CAUTI) were closely monitored to ensure process changes maintained or improved patient safety.

Initial positive results led to progressive expansion to all acute care and step-down care units across the hospital, totaling 27 units. Implementation across Cluster 1 expansion included seven units and followed the same phased approach using the EPIS Framework of implementation science of Exploration, Preparation, Implementation, and Sustainment.

#### 2.1.1. Exploration

The Exploration Phase of the continued initiative expansion required the continuation of relationship development among the VRCP team and impacted parties, including nurse leaders, frontline staff, and patients and families. Results and learnings from the pilot phase were a part of the exploration for the expansion of this initiative. The primary observable metric from the pilot phase was UAP retention, which demonstrated a positive change during and after implementation on pilot units. The secondary metric was self-reported improvements in team dynamics and collaboration by both UAP and RN roles via an anonymous virtual survey collected in REDCap^®^, a secure application for building and managing online surveys and databases headquartered in Nashville, TN, USA. These results, paired with the recognition that this initiative was feasible, provided an opportunity to expand into Cluster 1.

#### 2.1.2. Preparation

The Preparation Phase consisted of initial data collection and VRCP program socialization and training. Initial observations occurred from 12 August 2025 to 12 September 2026, where four VRCP team RNs were directly supported by the Principal Investigator (PI), a PhD-prepared RN who is trained in data collection and research and quality improvement methodology. The four RN observers of the VRCP team established IRR within the Cluster 1 units using the VRCP Reliability Checklist (see Supplementary Materials). Validity of the VRCP Reliability Checklist was completed through qualitative analysis of findings, regular collaborative meetings between RN observers, data analysts, leadership teams, and frontline staff. No statistical validation of the VRCP Reliability Checklist was completed in the scope of this quality-improvement initiative, as it was intended to reduce human error, support accuracy and quality, and ensure consistent performance.

The VRCP Reliability Checklist was built directly from the minimum standard expectation for communication, developed by consensus with frontline staff through shared governance processes. It supported observations of shift handover processes, including the date, time, and unit of observation, as well as the RN(s) and UAP(s) being observed and their patient workload. Three different points of communication may be observed, depending on the time of the observation. During handover observation, which was observed at 7 a.m. and 7 p.m., the VRCP Reliability Checklist observes the initiation and tools used during handover in addition to key patient safety information (name, diagnosis, code status, allergies, precautions), in addition to level of assistance support, psychosocial considerations, and body system needs to address any present neurological, cardiac, respiratory, gastrointestinal, genitourinary, and skin concerns. During planning observation, which was observed from 7 a.m. to 9 a.m. and 7 p.m.–9 p.m., observations were made to identify intentional care planning discussions in the RN–UAP dyad, including vital sign frequency and parameters, laboratory or glucose monitoring needs, ambulation and activity level, support needs for turns, intake and output monitoring needs, and the use of closed-loop communication. During review observation, which was observed from 3 a.m. to 7 a.m. and 3 p.m.–7 p.m., observations were made to identify existing items from the planning session that required updates or new patient care needs based on patient status, orders, or plan of care.

An electronic system for data reporting was built, allowing for comparisons across time and units for each key patient safety item and body system. Five unique measures—fall risk, level of assistance, toileting support, last turned time, and Q2 turn support—were identified as potential influencing factors for the nursing-sensitive quality indicators of falls with harm, pressure injuries, and catheter-associated urinary tract infections (CAUTI). Communication of each item scored a 1, while no communication of each item scored a 0, allowing for a total quality communication score of 0–5 for each observation.

All previously validated observation tools identified in the literature review required frontline staff participation, which was identified to be a potential barrier in accurate data collection due to organizational survey fatigue. Because of limited tool availability feasible for organizational use, a reliability checklist was identified by the PI and other researcher collaborators as the most appropriate option to gather data consistently. Process reliability checklists are common tools in high-reliability organizations, including healthcare environments, to measure adherence to standardized process change [20].

Additionally, the VRCP team attended leader meetings and collected baseline staff survey data related to RN–UAP teamwork and communication. This survey was developed in collaboration with the VRCP team, PI, frontline staff, and leaders. It collects responder-specific information including shift worked, unit, role, and years of experience. It also allowed participants to provide personal perspectives on team communication and opportunities for improvement. This survey was used to assess and improve VRCP processes for immediate integration into operational improvement in the QI initiative, rather than for hypothesis testing requiring validation.

Leader meetings provided an overview of upcoming process changes, which included standardized team communication processes, frequencies, and timelines. Process changes discussed included the transition to a complete UAP report using VRCP standardized communication tools at the start of shift, an intentional planning session for each patient between RN and UAP in the first two hours of the shift, and a review in the last four hours of shift focusing on incomplete tasks and changes in patient condition or plan.

VRCP socialization and training occurred from 15 September 2025 to 17 October 2025 and included baseline observations across the units, proactive rounding for concept socialization, formal team training, and a leader toolkit with resources to support the practice change. The PI calculated the minimum number of observations required to understand current baseline practice. It was decided by the PI and colleagues that 20% of the number of nurses employed by that unit would be sufficient to determine the number of observations completed in each unit, for a minimum goal of 79 observations across all Cluster 1 units. Mandatory formal team training was provided to all impacted staff, emphasizing communication, respect, and scope clarity alongside the change in process and new team expectations. Attendance at the team training was required for all staff and effectively managed by individual unit leaders to ensure attendance.

This four-hour team training incorporated adult learning principles and best practices for team training identified in the literature, such as interprofessional activities and development of role empathy [14]. The course was taught by NPD practitioners who specialize in ongoing professional education in healthcare environments. Course details reviewing the content areas, objectives, and interprofessional collaborative activities are available in Table 1. Course objectives were met through providing interprofessional collaborative opportunities to apply knowledge, including a gallery walk to understand universal relationship skills, completion of role-based puzzles to develop role empathy and understand scope of practice, video vignettes and formal debriefs to review current and future state of communication practice, and small group role play to demonstrate effective RN–UAP communication. Each objective and corresponding application activity accounted for approximately one hour of the course, including breaks between each content area. Online evaluations completed at the end of each course identified that learning objectives were successfully met by participants.

The UAP scope of practice within Tennessee and the organization was reviewed to ensure alignment and emphasis on UAPs working to the top of their scope (See Table 2). This training was founded in adult learning principles and used gallery walks, vignettes, and reflective dialog to allow learners to practice communication and delegation while engaging in role empathy in a psychologically safe and supportive environment.

#### 2.1.3. Implementation

The Implementation Phase included the VRCP initiative implementation and post-implementation support. The initiative implementation occurred from 13 October 2025 to 21 November 2025. The VRCP team supported unit leaders, observing and providing just-in-time support and coaching for staff. Unit leaders—including managers, assistant managers, and clinical leads—coordinated daily support for their units, with individuals assigned to be present and observe handover at each shift change daily for three weeks. Daily huddles were held by the VRCP team for all unit leaders during the first week of implementation from 13 to 17 October 2025, bi-weekly from 20 October 2025 to 7 November 2025, then weekly starting 10 November 2025. In these huddles, leaders reported successes, challenges, and needed support from the VRCP team. This allowed for immediate collaborative problem-solving. Three of the seven units had strong leader and RN engagement; however, across four of the seven units, leader and RN engagement was initially low. The units with lower leader and RN engagement required additional VRCP team support and resources during the entire implementation phase, including post-implementation support. Once the benefits of the program began to be realized, the initially lagging leaders took a more active and supportive role, and RN engagement increased, allowing for more effective implementation.

Post-Implementation support continued from 24 November 2025 to 12 December 2025, where formal and informal rounding occurred. Formal rounding was the structured observational process conducted during designated time frames when observers used the VRCP Reliability Checklist to evaluate adherence to SBARR handover and the Plan-Do-Review process. The Plan-Do-Review process is a safety reliability skill often used within high-reliability organizations. Formal observations included monitoring a predetermined number of interdisciplinary staff interactions to assess consistency and compliance. Informal rounding was less structured and occurred more frequently on an impromptu basis, emphasizing real-time engagement, providing immediate coaching, feedback, and mentorship to support practice improvement. Weekly meetings with leaders continued from 24 November 2025 to 12 December 2025, allowing for real-time feedback and unit leader support.

#### 2.1.4. Sustainment

The Sustainment Phase occurred from 22 December 2025 to 16 January 2026, and included continued formal and informal rounding. Monthly leader meetings, in addition to any individually requested support, continued, and leaders were responsible for developing and submitting unit-specific sustainment plans by the end of the sustainment phase. During this time, unit-specific observed behavior and qualitative feedback were shared with unit leaders and staff. A post-implementation survey to measure staff perception of teamwork and communication was collected, and leaders began taking additional ownership over the process change. The survey was available during a two-week period at the beginning of the sustainment phase and was available electronically for completion by all unit staff. The survey included four standardized questions and one role-specific question:

1. Do you attend a unit huddle at the beginning of your shift? Response options included Always, Sometimes, Never, or Unit does not have a huddle at shift change.
2. During report at shift change, do nurses involve care partners in the handover process? Response options included Always, Sometimes, or Never.
3. How often do you check in with your RN or UAP partner during a shift? Response options included 0 Times, 1 Time, 2 Times, or 3 or More Times.
4. Do you check in with your RN or UAP partner at the end of each shift? Response options included Always, Sometimes, or Never.

Role-specific questions were open-ended. RNs were asked, “As a nurse, what would you recommend to improve the teamwork between RNs and UAPs on your unit?” UAPs were asked, “As a care partner, what would you recommend to improve teamwork between RNs and UAPs on your unit?”

## 3. Results

Data analysis includes reporting of participant demographic data, retention data for UAP and RN roles, communication observations, teamwork perceptions, and quality measures. In addition to reporting raw numerical data, statistical analysis for quality communication observation scores—including chi-square and *p*-value—and quality measures—including observation and expected ratios, confidence intervals, and *p*-values—were completed using Microsoft^®^ Excel^®^ for Microsoft 365, headquartered in Washington, DC, USA. As data were collected for quality improvement purposes, some additional statistical analyses were unable to be completed due to the approach of data collection.

### 3.1. Participant Demographics

There were a total of 518 clinical staff who were part of Cluster 1 implementation: 180 UAPs, 335 RNs, and three unit nurse managers. This accounted for 100% of the clinical staff across seven acute care and step-down units in Cluster 1 (See Table 3). Across all units, UAPs had an average of 2.2 years of experience, RNs had an average of 6.0 years of experience, and Nurse Leaders had an average of 7.1 years of experience. This data does not account for those staff who were actively training as an RN resident or UAP trainee at the time of data retrieval.

### 3.2. Retention

Retention rate goals for both the UAP and RN roles were to increase retention by 1.5% compared to the FY25 baseline retention rates for each unit, for an RN retention goal of 83.4% and UAP retention goal of 67.5%. Pre-implementation comparison includes July–September 2025 and post-implementation from December 2025 to February 2026. During implementation, the Orthopedic Trauma unit changed locations and sizes, making historical data unavailable for comparison.

The retention rate goal was achieved overall for RNs, with a post-implementation rolling 12-month retention rate of 83.6% across all units, exceeding the predetermined goal of 83.4% (Table 4). The retention rate goal for UAPs was established at 67.5%. During the evaluation period, one participating unit—Myelosuppression and Stem Cell Transplant—experienced substantially higher apparent turnover than all other units. Review of Human Resources retention records determined that this increase was primarily attributable to internal mobility, specifically the planned transition of multiple UAPs into the organization’s Nursing Residency Program rather than voluntary resignation or organizational attrition. These reasons for separation were independently documented and verified by the unit’s Human Resources Business Partner as part of routine workforce monitoring and were unrelated to the quality-improvement initiative or other unit-culture factors.

Because unit-level retention metrics classify transfers to other organizational roles or departments as turnover, this measure does not distinguish workforce loss from internal career advancement. Given that the observed decrease in unit-specific retention reflected documented internal mobility rather than failure to retain employees within the health system, analyses were examined both including and excluding this atypical unit. When all participating units were included, the UAP retention goal was not achieved. Excluding the outlier unit, the post-implementation UAP retention rate increased to 70.7%, exceeding the predetermined goal (Table 5). Reporting both analyses provides transparency while acknowledging the limitations of unit-based retention metrics in organizations with opportunities for internal career progression.

### 3.3. Teamwork

#### 3.3.1. Observations of Communication

Communication observations were completed pre-implementation (80 observations) and post-implementation (85 observations), accounting for a minimum of 20% of the total number of frontline nurses employed within the seven implementation units (See Table 6). The standard at this organization is to provide report by exception, meaning that findings within normal limits (WNL) are not required to be verbalized during handover report. The handover was complete after review of all basic safety information and discussion of any items outside of organizationally identified WNL parameters. Items that were not discussed were verified by the observers to be WNL through patient documentation. If an aspect of communication was not discussed and documentation was also found to be outside of normal limits, it was identified as incomplete handover communication.

Notably, UAP concerns over the time required to complete patient report in the new workflow were identified as a potential barrier during team training. Across Cluster 1, pre-implementation report took on average 48 s or 0.8 min per patient. Post-implementation report took on average 126 s or 2.1 min per patient.

**Figure 1:**
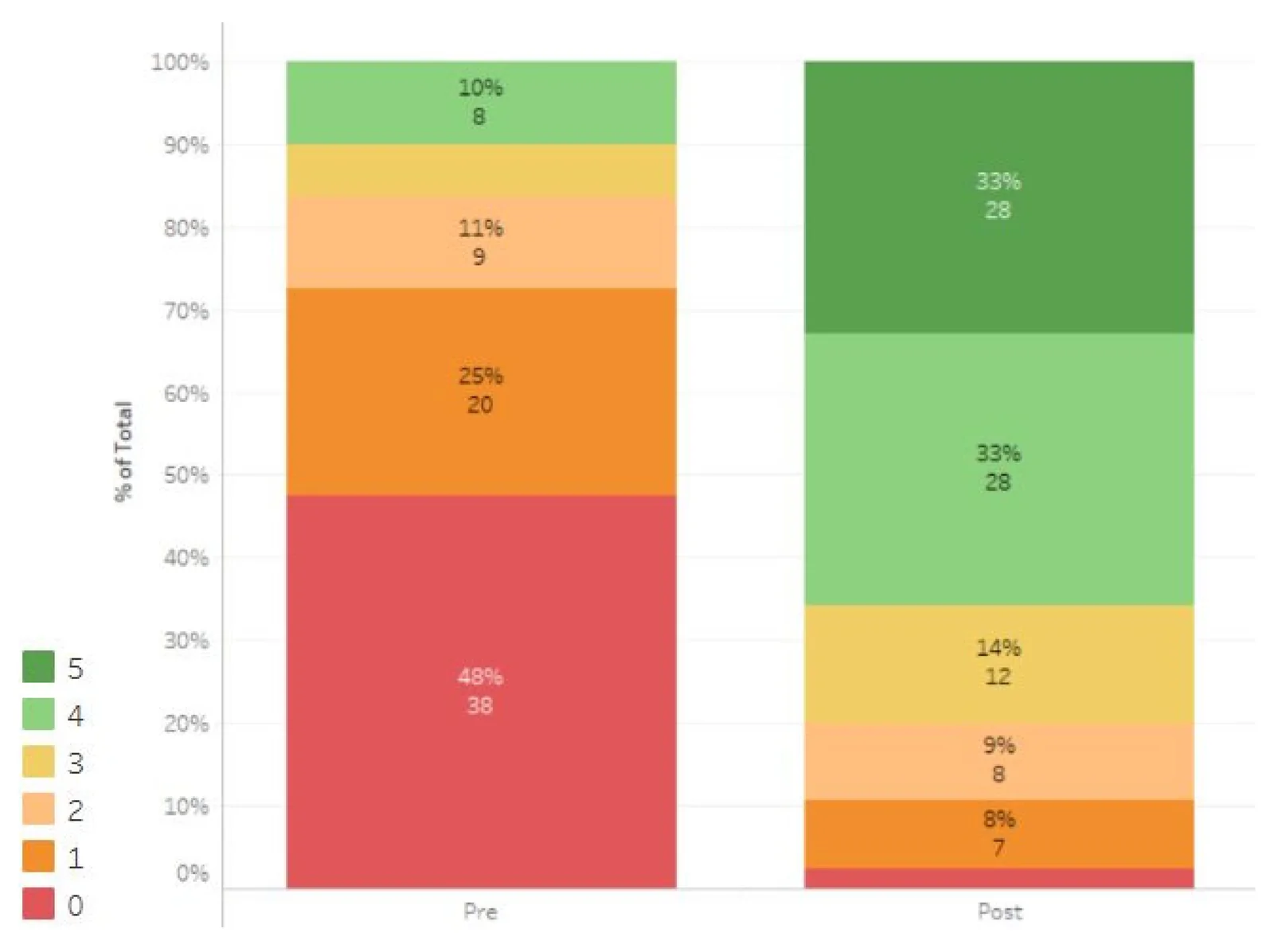
Quality score pre- and post-implementation.

Critical items for handover that addressed quality measures included fall risk status, bed rest or mobility needs, turn schedule, the last time turned, and toileting needs. Each observation tracked these components within the RN–UAP handover communication to provide an overall quality communication score (Table 7 and Figure 1). Pre-implementation, zero of these quality measures were discussed during report 48% of the time, one indicator was discussed 25% of the time, two measures were discussed 11% of the time, three measures were discussed 6% of the time, four measures were discussed 10% of the time, and five measures were discussed 0% of the time. After implementation, zero of these quality measures were discussed during report 3% of the time, one indicator was discussed 8% of the time, two measures were discussed 9% of the time, three measures were discussed 14% of the time, four measures were discussed 33% of the time, and five measures were discussed 33% of the time. Chi-square analysis demonstrated statistical significance with a chi-square of 80.73 and five degrees of freedom, as well as a *p*-value < 0.001.

The five observed points of communication that impacted the chosen quality measures of Falls, Pressure Injuries, and CAUTI were fall risk, activity level, turn schedule, last turn time, and bathroom support needs.

#### 3.3.2. Perception of Teamwork by Staff

A total of 145 staff members (109 RNs and 36 UAPs) across the seven Cluster 1 units participated in the pre-implementation survey. In those same units, 87 staff members (57 RNs and 30 UAPs) participated in the post-implementation survey, representing a lower overall response rate than at baseline. Item-level missing data were minimal, with only one unanswered perception item across all completed surveys. Responses to the optional open-ended question regarding opportunities to improve teamwork were not provided by all participants.

Survey responses were compared pre- and post-implementation periods and demonstrated improved communication between the RN–UAP dyad, as perceived by both RNs and UAPs. Responses to the same survey question in the pre-survey varied by role, with RNs generally perceiving greater levels of UAP collaboration, specifically in the handover process and at the beginning of each shift. Following implementation, responses from both RNs and UAPs reflected improved perceptions of collaborative communication with a reduction in the differences observed between professional groups (Table 8).

In response to the open-ended survey questions, RNs identified the importance of adjusting their reporting methods to improve consistency when communicating with the teams, the impact of pairing assignments to reduce the number of individuals within the RN–UAP teams, and the value of proactive planning for the shift (Table 8). UAPs reported that they felt more supported in speaking up within their teams, had a better appreciation and understanding of the complexity of the nursing role, and also identified the positive impact of reducing the number of individuals within the RN–UAP teams on their ability to communicate effectively (Table 9).

### 3.4. Quality

The nursing-sensitive quality measures identified as being positively impacted through better dyad communication were falls with harm, pressure injuries, and CAUTI. Intentional collaborative communication among the team is just one of many factors that may influence quality measures. Goals were derived based on the FY24 and FY25 data and trends, current trajectory, anticipated changes in patient volume and acuity, and intention to sustain quality improvement progress. National database of nursing quality indicators (NDNQI) national benchmarking is used to compare current quality to other large academic medical centers with >500 beds and a level of similar acuity, allowing leaders to continue to see opportunities for improvement and to drive unit-specific engagement in quality efforts. Across all implementation units collectively, the Q1–Q3 expected rates to achieve FY26 goals for falls with harm, pressure injuries, and CAUTI were exceeded (Table 10). FY26 Q1–Q3 falls with harm were lower than expected, with an observed-to-expected ratio of 0.93 (95% CI 0.66–1.31). This difference was not statistically significant (*p* = 0.753). FY26 Q1–Q3 pressure injuries were lower than expected with an observed-to-expected ratio of 0.39 (95% CI 0.22–0.66). This was statistically significant (*p* < 0.001). FY26 Q1–Q3 CAUTIs were lower than expected, with an observed-to-expected ratio of 0.67 (95% CI 0.28–1.60). This reduction was not statistically significant (*p* = 0.483).

## 4. Discussion

While implementation fidelity was high—as identified through adherence to project management timelines, maintaining expected initiative progression, and consistent frontline and leader support across all implementation science phases—it is important to note throughout the discussion that all metrics and results presented are multifaceted with many contributing factors. While the improvements across team dynamics and organizational goals are clearly demonstrated, it is possible that additional contributing factors, such as competing organizational quality improvement and workforce retention efforts, the overall effect and impact of the VRCP program. Therefore, the impact of the VRCP initiative may have been enhanced or reduced by external factors, and the changes noted were purely observed after the implementation period.

The VRCP quality-improvement initiative in an academic medical center demonstrates the impact of RN–UAP collaborative practice to support nursing team communication and organizational goals related to staff retention rates. Team communication was evaluated through observed handover processes within the RN–UAP dyad and frontline staff survey responses related to teamwork and communication within the dyad. RNs and UAPs were observed changing their communication practices to allow for proactive patient care planning and collaboration. Through streamlining patient care assignments and communication expectations, critical information became easier for RNs and UAPs to convey consistently and efficiently during handovers and within shifts. These findings align with previous research identifying improved communication skills, confidence, and handover completion using standardized communication tools [21,22]. Perhaps most relevant, previous findings also identified the need to implement role-specific communication tools to support sustained change [21].

Perception of communication and teamwork for both RNs and UAPs improved with the project implementation, indicating a better foundational understanding of communication expectations and processes. These improvements indicate stronger perceived value and collaboration within the team dyad, creating an environment that supports staff retention. This aligns with previous findings of standardized communication tools to positively impact interprofessional teamwork [15,23] and overall mental health for nurses [24]. These positive effects can be enhanced through communication team training as a method to increase psychological safety of team members and maintain patient safety as a team priority [25].

These observations were not just about performance, with implementation also showing a positive impact on quality measures and RN and UAP retention rates. Previous literature identifies opportunities for improved quality of care through more effective team communication [14,17]. Quality measures of the VRCP work support these findings, demonstrating incidents of pressure injuries, falls with harm, and CAUTI below expected rates and exceeding organizational goals. While multiple factors impact quality, intentional identification, discussion, and proactive planning within the RN–UAP dyad provides opportunities for concerns to be addressed early and preventative plans to be enacted; although only pressure injury reduction was statistically significant, every patient harm incident avoided is impactful for patients and families.

In considering retention, UAP retention rates were a significant concern in the organization, especially considering the increased number of UAPs used in the updated staffing models. Literature identifies that effective teamwork, often supported by communication, improves retention for UAPs and RNs [11,12,13,26]. The results of the VRCP work are concordant with the literature findings, with UAP retention increasing within most implementation units and meeting organizational goals. Similarly, RN retention rates improved after implementation, demonstrating the impact of better collaborative care and teamwork on RN retention.

The primary challenge of this QI initiative is sustainment after implementation. Lewin’s Change Theory demonstrates that there is a time after a change in process is made where consistent support is crucial to creating culture change for the new process to become the standard expectation, known as the stage of Refreeze, where the changes are solidified into organizational culture and structure [27]. In healthcare environments with many competing priorities, sustainment of new practices can be challenging. We have identified that engagement of each hospital unit nurse leader played a significant role in sustaining the new practices, which is consistent with the literature demonstrating leader support as a facilitator of frontline nurse engagement in quality-improvement initiatives [28]. Leaders who were engaged with VRCP, discussed the value of the work, and held staff accountable for the new expectations demonstrated consistent use of the new process and sustained unit improvements following initial implementation. Leaders who were more passive in the implementation required additional team support as units entered the sustainment phase of the project. The variable leader engagement was a challenge that remained throughout this work, regardless of the additional layers of sustainment support made available.

Because of these findings, leadership support was identified as a critical facilitating factor in the successful implementation of the VRCP initiative. This included exceptional support from executive leadership within the organization. Another facilitating factor was the continued accountability for this initiative through regular report-outs to executive leaders, unit leaders, and service line directors, shared governance for frontline staff, and the patient family advisory council. These layers of accountability ensured that wins and opportunities were shared with all impacted groups throughout the project and that barriers could be effectively and efficiently addressed.

### 4.1. Limitations

One limitation that occurred during the survey data collection from frontline staff was a reduction in survey participation, particularly among RNs from pre-implementation to post-implementation. Although the survey remained available for the same duration during both data collection periods, post-implementation recruitment relied more heavily on unit leadership to encourage participation because the VRCP team was concurrently supporting rollout on additional inpatient units. Consequently, recruitment efforts were less standardized across participating units and likely contributed to variability in response rates.

The lower post-implementation participation introduces the potential for nonresponse bias, as respondents may not fully represent the perceptions and experiences of all eligible frontline staff. Consequently, the observed improvements in teamwork and collaborative communication may have been under- or over-estimated if individuals with differing experiences were less likely to complete the survey. However, item-level missing data among completed surveys were negligible, with only one unanswered perception item, suggesting survey completeness was high among those who participated. Also, because surveys were completed anonymously and responses could not be linked across time points, changes in perceptions represent differences between two cross-sectional samples rather than within-person changes.

Another limitation of this intervention was the use of bundled interventions including communication tools, team training, ongoing observations and coaching, and leader support. This decision, while effective in supporting changed behavior and improved outcomes, created an environment where it was not possible to determine what individual interventions were most influential.

An additional limiting factor of this quality-improvement initiative was the increased time spent in handover report. Concerns from UAPs were centered around initiating tasks, such as vital signs, later than their previous standard and being unavailable to answer call lights during the longer report time. While report time did increase from 48 to 126 s per patient, the biggest shift to support this barrier came from requiring off-going staff to remain on the unit and engaged directly with their work until the end of their shift, rather than rushing through report and leaving earlier than scheduled. Also, units that adjusted the RN reporting structure and flow to prioritize the critical UAP needs were able to reduce time spent in UAP report and minimize stress related to time and task management.

### 4.2. Financial Considerations

The VRCP quality-improvement initiative is considered successful financially through cost avoidance and return on investment. Two primary areas are considered for cost avoidance with this work: reduced turnover and improved quality outcomes.

The average savings for each percent of improvement in nurse turnover is $328,400 [29]. From pre- to post-intervention, the retention rate for RNs increased by exactly 1%, for an RN cost savings of $328,400. While UAP turnover is not directly evaluated and available, the cost of medical assistant turnover is estimated to be $14,200 per individual [29]. With 130 UAPs participating across the five units with complete retention reporting and an overall unit retention rate improved by 1.8%, resulting in an estimated $33,230 saved. This does not account for all internal mobility within the listed units where organizational retention was maintained and therefore is likely an underestimation of the financial impact.

In considering cost avoidance through improved quality outcomes, the average cost to hospital organizations is $13,793 per CAUTI, $62,521 per fall with injury, and $20,900–$151,700 per pressure injury depending on the severity of injury [29]. When considering the current difference between observed and expected incidents, the VRCP initiative saved $34,482.50 on CAUTI, $156,302.50 on falls with injury, and $433,675 for the most conservative estimate on pressure injuries. This totals $624,460 saved through cost avoidance purely through improved quality measures.

These two financial considerations create an estimated total of $986,090 in cost avoidance through improved quality and retention of this single cluster, which accounts for approximately 25% of the total span of the initiative. The costs associated with the VRCP program in its entirety include the salaries of the nurse educators, nurse facilitators, observers, data analysts, and classroom materials across the entire grant cycle. All other costs associated with training time for staff were incurred directly by individual departments and were not centrally tracked and available for evaluation. The costs of the program development in its entirety were $890,000, indicating cost savings of just under $100,000, remembering that this accounts for approximately 25% of the total scope of the initiative, occurring in just the pre-implementation to post-implementation timeframe. These findings clearly demonstrate the financial benefit of the VRCP initiative in conjunction with other retention and quality improvement efforts, especially in scaling across a large organization for maximal impact and cost avoidance opportunity.

### 4.3. Future of the VRCP Initiative

This quality-improvement initiative continues to be disseminated across local, national, and international sectors to encourage other organizations with similar challenges to consider focusing on teamwork as an upstream factor to influence and improve retention of UAPs. In addition, other individual hospitals within the organization’s structure are identifying opportunities to implement this program into their own systems. This expansion work uses the foundations identified through the VRCP initiative and will be adjusted for their unique skill mixes, which include other job roles, titles, and scopes of practice.

This initiative helped the team to identify a gap in knowledge and understanding of best practices for patient assignment creation to maximize both RN and UAP communication while being responsive to patient workload and acuity. Next steps for progression of this work include additional literature reviews and practice changes to align patient assignments with VRCP processes.

Finally, sustainment of this initiative will be built into organizational culture through integration into the hospital’s structures. The formal team training remains mandatory for any new staff members of all 27 acute care and step-down units where this work was implemented, including integration of the foundational communication principles into nurse residency and UAP onboarding. Policies and procedures will be updated to reflect communication expectations for staff, and leader support tools will be developed and socialized for consistent understanding and practice across all levels of the organization. Finally, VRCP standard communication expectations will be integrated into the quality and safety department, including proactive rounding and follow-up after incident occurrences.

## 5. Conclusions

While challenges in RN–UAP communication are well-documented and expansive [10], this work demonstrates implementation of a three-pronged quality-improvement initiative to positively impact RN–UAP team functioning, staff retention, and patient care quality outcomes. Through team-based training, standardized communication tools, and reliable methods for observation and feedback, the VRCP program supported the achievement of meeting individual team and organizational goals.

## Figures and Tables

**Table 1:** Formal team training.

Topic	Objective	Interprofessional Collaborative Activity
Universal Relationship Skills	Identify one communication strategy they intend to use in their practice	Gallery walk
Roles & Responsibilities	Self-report enhanced understanding of RN and UAP role clarity and scope of practice	Role-based puzzles
RN-to-UAP Communication	Indicate an improved understanding of the significance of regularly scheduled check-ins throughout the shift	Video vignettes with structured debriefing
Fundamentals of Care	Successfully return to demonstrate appropriate delegation of UAP tasks	Role play guided by case studies

**Table 2:** Unlicensed assistive personnel scope of practice in the organization.

UAP Skills Within Scope of Practice			
Vital signs	Daily weight	Height	Abdominal girth
Intake & output	Blood glucose check	Lab specimen collection (phlebotomy, urine, stool)	Retention catheter insertion, maintenance, and removal
Straight catheterization	Bladder scan	Activities of Daily Living	Observe incentive spirometry
Pressure injury prevention bundle	Falls prevention bundle	Suicide precautions	Sequential compression device placement
Simple wound care	Peripheral IV removal	Emptying of drains, ostomy bags, and nephrostomy bags	Apply and remove restraints
Place telemetry	Initiate emergency procedures	Post-mortem care	

Notes: Scope of practice was determined in combination with Tennessee state law and organizational policy.

**Table 3:** Unit characteristics.

Specialty Unit	Level of Care	Beds	Average Daily Census	RN:UAP Staffing
Neurology and Epilepsy	Acute Care	27	26	5 RN:5 UAP
Adult Acute Care Medicine	Acute Care	18	16	4 RN:3 UAP
Orthopedic Trauma	Acute Care	35	35	7 RN:7 UAP
Myelosuppression & Stem Cell Transplant	Step Down	35	35	10 RN:5 UAP
Transplant and Surgical Care	Step Down	41	35	10 RN:7 UAP
Cardiovascular Progressive Care	Step Down	38	33	9 RN:6 UAP
Surgical Step Down	Step Down	31	29	8 RN:4 UAP

**Table 4:** Pre- and post-implementation rolling 12-month retention rates for RNs.

Unit	Pre-Implementation	Post-Implementation	Goal
Myelosuppression & Stem Cell Transplant	81.8%	87.1%	88.3%
Transplant & Surgical Care	81.9%	80.4%	74.6%
Neurology & Epilepsy	82.0%	80.1%	82.4%
Adult Acute Care Medicine	74.2%	76.0%	74.3%
Cardiovascular Progressive Care	80.5%	83.1%	87.6%
Surgical Step Down	95.1%	84.8%	93.0%
Total	82.6%	83.6%	83.4%

Notes: Goals were created based on a 1.5% improvement in retention from FY25. Orthopedic Trauma unit was not included in this metric.

**Table 5:** Pre- and post-implementation rolling 12-month retention rates for UAPs.

Unit	Pre-Implementation	Post-Implementation	Goal
Myelosuppression & Stem Cell Transplant	49.4%	33.0%	68.6%
Transplant & Surgical Care	75.4%	82.5%	61.0%
Neurology & Epilepsy	80.1%	84.1%	82.9%
Adult Acute Care Medicine	73.1%	74.0%	75.6%
Cardiovascular Progressive Care	57.8%	57.8%	68.0%
Surgical Step Down	58.3%	56.1%	49.9%
Total	65.7%	64.6%	67.5%
Total without Myelosuppression *	68.9%	70.7%	67.4%

Notes: Goals were created based on a 1.5% improvement in retention from FY25. Orthopedic Trauma unit was not included in this metric. * Removal of the Myelosuppression and Stem Cell Transplant from the data as a significant outlier due to external factors.

**Table 6:** Communication observations.

	Pre-Implementation (N = 80)		Post-Implementation (N = 85)	
Observation	Completion		Completion	
	(n)	%	(n)	%
Communication				
SBARR Tool Used	14	18	79	93
UAP Initiation of Report	67	84	81	95

Communication observations demonstrate clear behavior change and adoption of the quality improvement project into practice.

**Table 7:** Quality communication observations.

Quality Communication Score	Pre n (%)	Post n (%)
0	38 (47.5)	2 (2.4)
1	20 (25.0)	8 (8.2)
2	9 (11.3)	8 (9.4)
3	5 (6.3)	12 (14.1)
4	8 (10.0)	28 (32.9)
5	0 (0)	28 (32.9)

Note: Score distributions differed significantly between the pre- and post-intervention periods (X^2^ (5) = 80.73, *p* < 0.001).

**Table 8:** Survey responses (frequency of selecting “always”).

	RN Responses		UAP Responses	
Question	Pre (%)	Post (%)	Pre (%)	Post (%)
Do you attend a unit huddle at the beginning of your shift?	94	93	87	90
During report at shift change, do RNs involve UAPs in the handover process?	25	33	10	30
Do you directly communicate needs with your UAP or Nurse at the start of your shift?	49	68	21	67
Do you check in with your partnered RN or UAP at the end of each shift?	27	33	38	40

**Table 9:** Survey responses: direct quotes from staff on post-implementation teamwork perceptions.

Role	Statement
UAP	Everyone is starting to help more. We speak more during shift. It has changed my perspective of what nurses do. Now we know how to help each other.
RN	I feel that we always know what the plan is, and it allows better communication between us through the shift. There is better communication, respect, and teamwork. The best teamwork happens when nurses have one UAP to work with, and the UAP only has minimal nurses.

**Table 10:** Number of quality incidents across all implementation units.

Quality Measure	FY 24	FY 25	FY 26 Goal	FY26 (Q1–Q3) Expected	FY26 (Q1–Q3) Actual	Difference	O:E Ratio	95% CI	*p*-Value
Falls with harm	48	48	46	34.5	32	−2.5	0.93	0.66–1.31	0.753
Pressure Injuries	74	32	45	33.75	13	−20.75	0.39	0.22–0.66	<0.001
CAUTI	9	9	10	7.5	5	−2.5	0.67	0.28–1.60	0.483

Notes: Patient care quality measures related to falls with harm, pressure injuries, and CAUTI development demonstrate outcomes exceeding organizational goals.

## Data Availability

Internal datasets are not publicly available due to confidentiality restrictions related to sensitive organizational information. Quality-related measures are (or will be) available via federal quality dashboards and national healthcare transparency registries. Additional de-identified/aggregated data may be shared upon reasonable request subject to institutional approval.

## Supplementary Materials

The following supporting information can be downloaded at: https://www.mdpi.com/article/10.3390/nursrep16080265/s1, VRCP Reliability Checklist.

## Public Involvement Statement

There was no public involvement in any aspect of this research.

## Guidelines and Standards Statement

This manuscript was drafted against the SQUIRE 2.0 for quality improvement in healthcare.

## Abbreviations

The following abbreviations are used in this manuscript:

CAUTI	Catheter-Associated Urinary Tract Infection
CP	Care Partner
FY	Fiscal Year
IRR	Inter-Rater Reliability
IRB	Institutional Review Board
NPD	Nursing Professional Development
NDNQI	National Database of Nursing Quality Indicators
RN	Registered Nurse
UAP	Unlicensed Assistive Personnel
SBARR	Situation–Background–Assessment–Recommendation–Response
VRCP	Vanderbilt RN–Care Partner Partnership
WNL	Within Normal Limits
AACN	American Association of Colleges of Nursing
