# Supplementary material for: Standardized Communication Across Interprofessional Teams: A Quality-Improvement Initiative to Support Nursing Staff Retention
Source: Nurs Rep. 2026 Jul 30;16(8):265. doi: 10.3390/nursrep16080265 (PMC13516107; doi:10.3390/nursrep16080265)
Supplement: Supplementary file 1 [file nursrep-16-00265-s001.zip › nursrep-4375492-supplementary.pdf]

Unit: ___________________ Date: ______________________

Start Time: _______________ End Time: __________________

RN/CP: _________________ Years Exp: __________________

RN: ___________________ Years Exp: __________________

CP: ___________________ Years Exp: __________________

RN # of Pts: ___________ CP # of Pts: _________________

***Italic Measures are the Quality Measures we've identified for this project*

**RN to CP Shift Report Process Checklist (Handover)**	Yes	No
1. CP Initiates Report from RN/CP		
2. CP SBARR Tool Utilized		
3. Is the tool a handwritten SBARR tool or the pre-printed SBARR form?		
Report Includes the following:		
Room #		
Patient Name		
Diagnosis		
Code Status		
Allergies		
Precautions		
Neuro Section		
a. AxO or Confused		
c. HOH		
d. Vision		
e. Fall Risk		
**Level of Assist Section**		
a. Level of Assist		
c. Bed Alarm		
d. Assistive Device		
**Cardiac Section**		
a. Monitored		
b. Travel off?		
c. Device- VAD/Pacemaker		
d. Transplant		
**Respiratory Section**		
a. Oxygen Needs		
**GI Section**		
a. Diet		
b. Diet Restrictions		
**GU Section**		

a. Continent		
b. BRP/Urinal/BSC/Hat/Purewick/Condom/Foley/Bedpan/Ostomy		
**Skin Section**		
a. Wounds/Drains/Tubes		
b. Lines (Central, PICC, Port, PIV)		
c. Pressure Ulcers		
**Psychosocial Section**		
a. Planned Procedures		
b. Planned Discharge		

**Planning Observations**	Yes	No	N/A
**WAS A PLAN MADE? (credit is allocated based on this question)**			
1.VS Q____ Hours			
2. Reporting Parameters if outside of norm:			
3. Labs Due at:			
4. Accuchecks			
5. Ambulation @:			
6. Activity			
7. Toileting			
8. Bed Rest			
9. *Last Turned:*			
10. Q2 Turns			
11. Intake/Output Q___ Hours			
12. Closed Loop Communication Used			

**Reviewing Observations**	Yes	No	N/A
**WAS A REVIEW DONE? (credit is allocated based on this question)**			
1.VS Q____ Hours			
2. Reporting Parameters if outside of Norm:			
3. Labs Due at:			
4. Accuchecks			
5. Ambulation @:			
6. Activity			
7. Toileting			
8. Bed Rest			
9. *Last Turned:*			
10. Q2 Turns			
11. Intake/Output Q___ Hours			
12. Closed Loop Communication Used			
